# Double Jeopardy: Lamotrigine-Induced Drug Reaction With Eosinophilia and Systemic Symptoms (DRESS) Complicated by Valproate Hepatotoxicity

**DOI:** 10.7759/cureus.101034

**Published:** 2026-01-07

**Authors:** Ashna Afrin, Anees C K, Melvin C Anto, Sruthakeerthi R.N

**Affiliations:** 1 Internal Medicine, Dr. Moopen's Medical College, Wayanad, IND

**Keywords:** anti-seizure drug, drug reaction with eosinophilia and systemic symptoms (dress), hepatotoxicity, lamotrigine-associated drug reaction, valproate toxicity

## Abstract

A 17-year-old boy with primary generalized epilepsy developed a high fever, a widespread rash, swollen lymph nodes, and an enlarged liver and spleen a few weeks after starting lamotrigine. Blood tests revealed eosinophilia and elevated liver enzymes, meeting the Registry of Severe Cutaneous Adverse Reaction (RegiSCAR) criteria for drug reaction with eosinophilia and systemic symptoms (DRESS) syndrome. Lamotrigine was discontinued, and corticosteroid therapy was initiated, while antiepileptic treatment with valproate and levetiracetam was continued. Although his fever and rash resolved, liver function worsened, indicating valproate-induced hepatotoxicity. The valproate dose was reduced, resulting in gradual recovery, and the patient became completely well within days. This case highlights the challenge of recognizing overlapping drug reactions in patients on multiple antiepileptic medications. Awareness of the lamotrigine-valproate interaction, close monitoring, timely withdrawal of the offending drug, and prompt corticosteroid therapy are essential to ensure full recovery in such complex adverse drug reactions.

## Introduction

Drug reaction with eosinophilia and systemic symptoms (DRESS) is a rare but potentially life-threatening hypersensitivity reaction that typically develops two to eight weeks after initiation of the causative drug. It presents with fever, rash, lymphadenopathy, eosinophilia, and internal organ involvement, most commonly affecting the liver [1]. Among the many causative agents, aromatic anticonvulsants such as lamotrigine and carbamazepine are frequent triggers [1].

Lamotrigine-induced DRESS poses additional diagnostic and therapeutic challenges when used in combination with valproate. Valproate inhibits lamotrigine glucuronidation, thereby increasing its serum concentration and half-life, which in turn elevates the risk of hypersensitivity reactions [2,3]. In addition, valproate can independently cause idiosyncratic hepatotoxicity through mitochondrial dysfunction and inhibition of β-oxidation of fatty acids [4].

Because both lamotrigine-induced DRESS and valproate hepatotoxicity can present with rash, fever, and hepatic dysfunction, identifying the culprit drug may be difficult. This report describes a young male with lamotrigine-induced DRESS complicated by valproate-associated hepatotoxicity, highlighting the challenge of distinguishing overlapping toxicities and the importance of careful, stepwise evaluation when multiple antiepileptic agents are used.

## Case presentation

A 17-year-old boy with primary generalized epilepsy, on multiple antiepileptic drugs, such as levetiracetam 750 mg twice daily, valproate 500 mg twice daily, zonisamide 100 mg twice daily, and lamotrigine 100 mg twice daily, presented with a 10-day history of high-grade fever and a two-day history of diffuse erythematous rash. Lamotrigine had been introduced six weeks earlier.

On admission, he was febrile (103 °F) with a pulse of 84 bpm, blood pressure of 130/80 mm Hg, respiratory rate of 18 cpm, and oxygen saturation of 96% on room air. Examination revealed a generalized morbilliform rash over the trunk and limbs, bilateral cervical and axillary lymphadenopathy, and mild hepatosplenomegaly. Daily variations in temperature, pulse, blood pressure, respiratory rate, oxygen saturation, and hematologic parameters are summarized in Table 1.

**Table 1:** Vitals and hematological parameters This table summarizes daily vital signs and hematological findings during admission. The peak temperature, mild tachycardia, leukocytosis, and eosinophilia reflect the systemic inflammatory response typical of DRESS. Improvement paralleled corticosteroid therapy and withdrawal of the offending agent. DRESS, drug reaction with eosinophilia and systemic symptoms

Parameter	Patient value	Reference range	Units/notes
Temperature	103	97-99	°F
Pulse rate	84	60-100	bpm
Blood pressure	130/80	<140/90	Mm Hg
Respiratory rate	18	12-20	cpm
SpO₂	96	≥95	% (room air)
Hemoglobin	13	13-17	g/dL
Total leukocyte count	20,900	4,000-11,000	/µL
Neutrophils	36	40-75	%
Lymphocytes	52	20-45	%
Eosinophils	11	1-6	%
Platelet count	3.0 × 10⁵	1.5-4.0 × 10⁵	/µL
Ultrasound abdomen	Hepatosplenomegaly	-	-

Laboratory findings showed hemoglobin 13 g/dL, total leukocyte count 20,900/µL (neutrophils 36%, lymphocytes 52%, eosinophils 11%), and platelets 3.0 × 10⁵/µL. Peripheral smear demonstrated moderate eosinophilia and reactive lymphocytosis. Liver enzymes were mildly elevated (serum glutamic oxaloacetic transaminase (SGOT) 160 U/L, serum glutamate pyruvate transaminase (SGPT) 184 U/L), and ultrasonography confirmed hepatosplenomegaly. Based on these features and a Registry of Severe Cutaneous Adverse Reaction (RegiSCAR) score ≥5, a diagnosis of lamotrigine-induced DRESS was made.

Lamotrigine was discontinued, and intravenous dexamethasone 4 mg twice daily was initiated. On day 5, the patient experienced a single seizure, after which lacosamide was added for better seizure control. Fine-needle aspiration cytology of the cervical lymph node revealed reactive lymphoid hyperplasia.

Although the fever and rash gradually subsided, liver enzyme levels rose sharply (Table 2), with SGOT at 1610 U/L and SGPT at 2860 U/L.

**Table 2:** Serial liver enzyme trends This table shows serial hepatic enzyme values, indicating a hepatocellular pattern of injury. The marked rise in AST and ALT with normal bilirubin suggests valproate-related toxicity. Rapid decline after valproate dose reduction confirms temporal improvement. ALT, alanine transaminase; AST, aspartate aminotransferase; SGOT, serum glutamic oxaloacetic transaminase; SGPT, serum glutamate pyruvate transaminase

Date	SGOT (AST)	SGPT (ALT)	Reference range (U/L)
September 9, 2025	122	828	0-40 (AST); 0-41 (ALT)
September 10, 2025	800	1,500	-
September 11, 2025	1,610	2,860	-
September 13, 2025	1,266	1,810	-

In the absence of worsening rash or eosinophilia, and with continued use of valproate, known for dose-related hepatotoxic potential, especially in combination with enzyme-inhibiting drugs such as lamotrigine, the pattern suggested valproate-induced hepatotoxicity rather than progression of DRESS.

**Figure 1:**
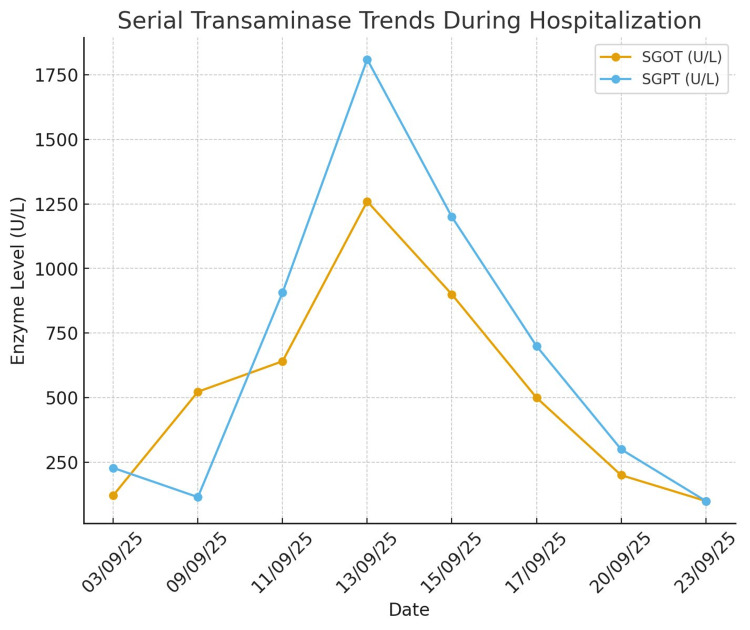
Serial transaminase trends during hospitalization Line graph depicting SGOT and SGPT trajectories during hospitalization. The continued rise in enzymes despite clinical improvement of DRESS indicates ongoing valproate-induced hepatocellular injury, while the sharp decline after dose reduction demonstrates biochemical recovery. DRESS, drug reaction with eosinophilia and systemic symptoms; SGOT, serum glutamic oxaloacetic transaminase; SGPT, serum glutamate pyruvate transaminase

Accordingly, the valproate dose was reduced to 300 mg twice daily, and levetiracetam was increased to 1.5 g twice daily. Over the following days, transaminase levels progressively declined, and systemic symptoms resolved completely by day 11 (Figure 1).

He was discharged on a tapering course of oral corticosteroids, along with levetiracetam 1.5 g twice daily, zonisamide, and lacosamide. At two-month follow-up, he remained clinically well, with complete normalization of liver enzymes and stable seizure control on the adjusted regimen.

## Discussion

DRESS is a rare yet potentially life-threatening hypersensitivity reaction that typically develops within two to eight weeks of exposure to the offending drug [1]. It is characterized by fever, rash, lymphadenopathy, eosinophilia, and internal organ involvement, most commonly affecting the liver. Among anticonvulsants, lamotrigine is a well-recognized cause, with an estimated incidence of 1 in 1,000-10,000 exposures [2]. The risk of DRESS increases significantly when lamotrigine is used in combination with valproate, as valproate inhibits lamotrigine metabolism via glucuronidation, thereby prolonging systemic exposure and potentiating immune sensitization [3,4].

In our patient, the temporal relationship between lamotrigine initiation and symptom onset, along with fever, rash, lymphadenopathy, eosinophilia, and transaminitis, fulfilled the RegiSCAR criteria for DRESS. Prompt withdrawal of lamotrigine and initiation of corticosteroids led to early defervescence and improvement in rash, supporting its causal role. However, the continued and marked rise in transaminases despite clinical improvement suggested a second hepatic insult. This prompted consideration of valproate-induced hepatotoxicity, a known but unpredictable adverse reaction associated with idiosyncratic mitochondrial injury.

The diagnosis of valproate-induced liver injury was supported by several features. First, the biochemical pattern was hepatocellular, with disproportionately elevated AST and ALT (peaking at 1610 U/L and 2860 U/L, respectively) but without hyperbilirubinemia, typical of valproate hepatotoxicity [5-7]. Second, enzyme elevation persisted despite clinical resolution of DRESS features and declined rapidly after valproate dose reduction, indicating a strong temporal and causal relationship. Third, no alternative causes, such as viral hepatitis, autoimmune hepatitis, or sepsis, were identified. Finally, literature reports that lamotrigine-valproate combination therapy may amplify hepatic vulnerability through metabolic interference and mitochondrial stress [8,9].

Valproate hepatotoxicity is believed to result from inhibition of mitochondrial β-oxidation, leading to accumulation of toxic metabolites, oxidative stress, and microvesicular steatosis [5,6]. Although it can occur at therapeutic doses, the risk increases in the presence of concomitant enzyme-inhibiting drugs, rapid dose escalation, or polytherapy. Recognition of this idiosyncratic mechanism is critical because the clinical picture may overlap with hepatic involvement in DRESS, potentially delaying diagnosis.

Systemic corticosteroids remain the cornerstone of therapy in moderate-to-severe DRESS, particularly when hepatic or other visceral organs are involved. They promote rapid improvement in fever, rash, and inflammation and reduce relapse rates when tapered gradually [10,11]. In this case, intravenous dexamethasone resulted in rapid improvement, and subsequent tapering prevented recurrence. The steady decline in liver enzymes after valproate reduction further reinforced the dual-drug etiology and the importance of ongoing biochemical monitoring.

This case illustrates how overlapping toxicities from lamotrigine and valproate can complicate both diagnosis and management. The sequential improvement after lamotrigine withdrawal and valproate reduction highlights the importance of considering multiple drug interactions in patients on antiepileptic polytherapy. Clinicians should maintain a high index of suspicion when hepatic enzymes continue to rise despite clinical improvement in DRESS. Careful titration, slow cross-switching, and early discontinuation of the offending drug(s) remain key to a favorable outcome.

## Conclusions

DRESS is a potentially life-threatening condition in which early recognition and prompt withdrawal of the offending drug are critical to preventing clinical deterioration. In this case, the coexistence of lamotrigine-induced DRESS with subsequent valproate-associated hepatotoxicity created diagnostic uncertainty and required stepwise clinical reassessment.

The sequential improvement after discontinuation of lamotrigine, followed by biochemical recovery after valproate dose reduction, highlights the importance of closely monitoring liver function and reconsidering causality when abnormalities persist. A structured, stepwise approach to antiepileptic polytherapy, combined with judicious corticosteroid use, may help prevent delays in diagnosis and improve outcomes in similar cases.
